# Assessing trade-offs in large marine protected areas

**DOI:** 10.1371/journal.pone.0195760

**Published:** 2018-04-18

**Authors:** Tammy E. Davies, Graham Epstein, Stacy E. Aguilera, Cassandra M. Brooks, Michael Cox, Louisa S. Evans, Sara M. Maxwell, Mateja Nenadovic, Natalie C. Ban

**Affiliations:** 1 School of Environmental Studies, University of Victoria, Victoria, British Colombia, Canada; 2 Environmental Change and Governance Group, School of Environment, Resources and Sustainability, University of Waterloo, Waterloo, Ontario, Canada; 3 The Leonard and Jayne Abess Center for Ecosystem Science and Policy, University of Miami, Miami, Florida, United States of America; 4 Stanford University, Emmett Interdisciplinary Program in Environment and Resources, Stanford, California, United States of America; 5 Environmental Studies Program, Dartmouth College, Hanover, New Hampshire, United States of America; 6 Geography, College of Life and Environmental Sciences, University of Exeter, Exeter, United Kingdom; 7 Australian Research Council Centre of Excellence for Coral Reef Studies, James Cook University, Townsville, Queensland, Australia; 8 Department of Biological Sciences, Old Dominion University, Norfolk, Virginia, United States of America; 9 Duke University Marine Laboratory, Nicholas School of the Environment, Duke University, Beaufort, North Carolina, United States of America; University of Sydney, AUSTRALIA

## Abstract

Large marine protected areas (LMPAs) are increasingly being established and have a high profile in marine conservation. LMPAs are expected to achieve multiple objectives, and because of their size are postulated to avoid trade-offs that are common in smaller MPAs. However, evaluations across multiple outcomes are lacking. We used a systematic approach to code several social and ecological outcomes of 12 LMPAs. We found evidence of three types of trade-offs: trade-offs between different ecological resources (supply trade-offs); trade-offs between ecological resource conditions and the well-being of resource users (supply-demand trade-offs); and trade-offs between the well-being outcomes of different resource users (demand trade-offs). We also found several divergent outcomes that were attributed to influences beyond the scope of the LMPA. We suggest that despite their size, trade-offs can develop in LMPAs and should be considered in planning and design. LMPAs may improve their performance across multiple social and ecological objectives if integrated with larger-scale conservation efforts.

## Introduction

Society increasingly expects protected areas to achieve a diverse set of objectives, ranging from conserving biodiversity to improving local livelihoods and mitigating the impacts of climate change [1]. Yet with increased expectations comes a challenge: it is rarely possible for protected areas to be successful across all these domains. Although there is a desire to characterize protected areas as universally beneficial for the environment and people (i.e., “win-win”), conservation initiatives involving multiple parties and limited resources often involve trade-offs as the norm, rather than the exception [2]. Trade-offs can occur among management objectives, ecosystem services, stakeholders, and values [2, 3] and involve “gains for one ecosystem service or group of people, resulting in losses for others” [3]. The desirability of different objectives, services or values is subjective and can vary with the perspective of different stakeholders. Trade-offs can be explicit management decisions, or arise as an unintended consequence of conservation actions, and can be understood in disparate ways, influenced by social norms and life experiences [2]. The evaluation of trade-offs is important because it facilitates a more complete consideration of the impacts of conservation initiatives, the lack of which can alienate important partners and reduce support for conservation [4].

International conservation policies, such as the Convention on Biological Diversity, have supported the expansion of the global protected area network over the last decade (e.g., Aichi Biodiversity Targets [5]), including large marine protected areas (LMPAs) [6]. Expectations for the social and ecological performance of these large areas, some of which exceed one million km^2^, are immense. For example, they have been described as “our best hope for arresting the global decline in marine biodiversity” [7]. It has been hypothesized that LMPAs may be able to avoid livelihood trade-offs that frequently occur in smaller MPAs because they are better able to accommodate resource use within their boundaries and thus have less impact on communities, and also have greater ecological benefits including protecting wide-ranging species [7–10]. However, there is considerable debate concerning the benefits of LMPAs (e.g., [11, 12]), partially arising from an absence of systematic evaluations of their performance (but see [13]). Although insights regarding trade-offs in smaller MPAs may be informative, LMPAs have different management challenges than smaller MPAs, including a greater diversity of habitats [14], multiple agencies with overlapping statutory responsibilities and jurisdictions, a more diverse and often remote constituency, and enforcement challenges [12, 15–17]. Therefore there is a need to evaluate the occurrence of trade-offs in LMPAs to identify opportunities to improve their effectiveness across multiple outcomes.

Despite calls for the analysis of trade-offs in conservation [2], empirical studies involving multiple outcomes are rare (but see: [13, 18, 19]). Trade-offs can occur within ecological or social systems or between them, and can be assessed through a variety of approaches, including theories and models [20], empirical evaluations of specific types of trade-offs (e.g., temporal trade-offs [21]) and between different beneficiaries [22]. Mouchet and colleagues [23] drew upon the trade-off frameworks used in the Millennium Ecosystem Assessment [24] and the Economics of Ecosystems and Biodiversity [25] to develop a typology identifying three general types of trade-offs that emerge in social-ecological systems. First, supply trade-offs are those which involve two or more ecological resources or ecosystem services. An example is the rapid change in relative abundance of groundfish (i.e. Atlantic Cod) and invertebrates (i.e. shrimp, crab) along the coast of Newfoundland in Canada [26]. Second, supply-demand trade-offs are those involving an ecological resource or ecosystem service and the well-being benefits they provide to communities or stakeholders (i.e., between the ecological and social outcomes). Overfishing is a classic example of a supply-demand trade-off in which livelihood benefits are maintained or enhanced at the expense of resource conditions. Third, and finally, demand trade-offs are those involving social outcomes among different groups of stakeholders. For instance, conservation initiatives may provide benefits for recreational fishers or tourism operators, but might displace fishers that have traditionally relied upon those areas for their livelihoods.

In this paper we evaluate whether trade-offs occur in LMPAs. We assess several social and ecological outcomes across a set of well-established LMPAs using a consistent coding approach. Trade-offs were classified using the typology from Mouchet et al. [23] and then plausible trade-off mechanisms were evaluated to elucidate how trade-offs may be occurring. Finally, we conclude with a discussion of opportunities for addressing different types of trade-offs.

## Methods

### Selection of case-studies

LMPAs were selected based on four criteria: 1) biodiversity conservation as a primary goal; 2) large: defined as >10,000km^2^ (several magnitudes larger than the median size of MPAs (3.3km^2^; [6])); 3) five years of active management: defined as having a management plan and some implementation for at least five years; and 4) sufficient data on outcomes. We identified LMPAs that met our first three criteria from MPAtlas.org [27], and then conducted a preliminary literature search to determine whether there was evidence of management actions (i.e. environmental monitoring, enforcement). We considered there to be sufficient data for coding outcomes when there were published peer-reviewed or grey literature sources that assessed ecological and/or social outcomes. Globally, 16 MPAs met the first two criteria, four of which were later excluded because they either lacked active management or adequate data on outcomes (Greenland National Park, Dominican Republic Marine Mammal Sanctuary, Franz Josef Land, Pelagos Sanctuary). Our final sample of 12 MPAs range in size from 11,859 km^2^ (Raja Ampat MPA Network) to 362,073 km^2^ (Papahānaumokuākea Marine National Monument), and in age from 10 years (Raja Ampat MPA Network) to more than 40 years (Svalbard Eastern Nature Reserves and Great Barrier Reef; S1 Fig).

### Coding of cases

We used the Social-Ecological Systems Meta-Analysis Database (SESMAD) [28] to provide a consistent approach for coding outcomes across the 12 LMPAs. SESMAD is a relational database based upon the social-ecological systems framework [29] that uses mostly categorical and ordinal variables to describe components of a social-ecological system and enable comparisons across cases where different metrics might be used. For each LMPA, we focused on five outcomes (Table 1): three outcomes associated with the ecological system (changes in an ecosystem health, a target fishery, and a key migratory species), and two outcomes associated with resource users (changes in the well-being of a user associated with ecosystem health, changes in the well-being of a user associated with the target fishery). Additional information concerning methods and coding are found in [13], and coded cases can be viewed at: https://sesmad.dartmouth.edu/ses_cases.

**Table 1:** The three main components of the social-ecological system and the five outcomes measured in this study.

Outcome	Social-ecological system component	Definition	Possible values
Ecosystem health	Resource (ecological system)	What is the change in ecosystem health over the time frame assessed?	Increasing; stayed the same/mixed effects; decreasing
Fishery	Resource (ecological system)	What is the change in the fishery over the time frame assessed?	Increasing; stayed the same/mixed effects; decreasing
Migratory species	Resource (ecological system)	What is the change in the migratory species over the time frame assessed?	Increasing; stayed the same/mixed effects; decreasing
Well-being of user related to ecosystem resource	Resource user	What is the change in the well-being of the user associated with the ecosystem health resource over the time frame assessed?	Increasing; stayed the same/mixed effects; decreasing; NA (no user)
Well-being of user related to fishery	Resource user	What is the change in the well-being of the user associated with the fishery resource over the time frame assessed?	Increasing; stayed the same/mixed effects; decreasing; NA (no user)

We conducted a detailed literature review of peer-reviewed and grey literature for each LMPA to identify potentially relevant components across the social-ecological system. Natural components (i.e. fish, migratory species, and indicators for ecosystem health) were selected for coding based upon: 1) their influence at the scale of the LMPA; 2) explicit mention of the natural component in the LMPA management plan or governance guidance; 3) data availability (i.e., changes in the natural component have been documented), and; 4) where multiple options existed, we selected components that would be expected to respond to governance. For instance, in Macquarie Island Marine Reserve, we selected Royal Penguins as an indicator of ecosystem health because they are a higher trophic level species and breed exclusively on islands within the Reserve. User groups, meanwhile, were determined by considering whether there was a group of actors that derived a non-trivial fraction of their livelihood benefits, whether directly or indirectly from the selected components. For instance, fishers in the Great Barrier Reef clearly derive livelihood benefits from reef fish, but also depend indirectly upon coral cover to maintain the supply of reef fish. In contrast, the livelihoods of fishers in the Heard Island and McDonald Island Marine Reserve are not substantially related to the health and abundance of King Penguins. The specific components coded for each case are identified in Table 2.

**Table 2:** Details on the components coded for each large marine protected area.

MPA Name	Fisheries Interaction		Ecosystem Health Interaction		Migratory Species Interaction	Time period assessed (snapshot)
	Fishery	User group	Indicator	User group	Migratory Species	
Cenderawasih Bay National Park	Reef fish	Artisanal fisher	Coral cover	Artisanal fisher	Green turtle	2002–2015
Central California National Marine Sanctuary	Groundfish habitat	Commercial fisher	Rocky intertidal	Researchers	Humpback whale	1992–2015
Galapagos Marine Reserve	Brown sea cucumber	Artisanal fisher	Sharks	Tourism	Green turtle	1998–2015
Great Australian Bight Marine Park	Southern Bluefin tuna	Commercial fisher	Australian Sealion	Commercial fisher	Southern right whale	2000–2012
Great Barrier Reef Marine Park	Reef fish	Commercial fisher	Coral cover	Commercial fisher	Green turtle	2005–2015
Heard Island and McDonald Island	Patagonian toothfish	Commercial fisher	King penguin	NA	Light mantled albatross	2002–2012
Macquarie Marine Reserve	Patagonian toothfish	Commercial fisher	King penguin	NA	Light mantled albatross	2001–2015
Papahānaumokuākea Marine National Monument	Lobster	NA	Trophic density	NA	Green turtle	2006–2015
Raja Ampat Marine Protected Area Network	Reef fish	Artisanal fisher	Coral cover	Artisanal fisher	Green turtle	2009–2015
Seaflower Marine Protected Area	Groupers (6 species)	Artisanal fisher	Coral cover	Artisanal fisher	Green turtle	2005–2015
Svalbard Eastern Nature Reserves	Shrimp	Commercial fisher	Polar bear	Tourism	Black-legged kittiwake	2002–2012
Wakatobi Marine Park	Reef fish	Artisanal fisher	Coral cover	Artisanal fisher	Green turtle	2008–2015

Changes in resource conditions and well-being were coded using SESMAD protocols [28] to explore the implications of a wide range of social, ecological and institutional factors for sustainability (11), and generate insights about potential trade-offs. Each LMPA was assessed for a specific time-period (or ‘snapshot’) in which the governance structure remained relatively stable (i.e., no major re-zoning), while outcomes were coded as changes in resource conditions or well-being over this time period. All outcomes were ordinal with three possible values, and were recorded as missing (NA) in the absence of a user group (Table 1).

### Evaluation of trade-offs

**Fig 1:**
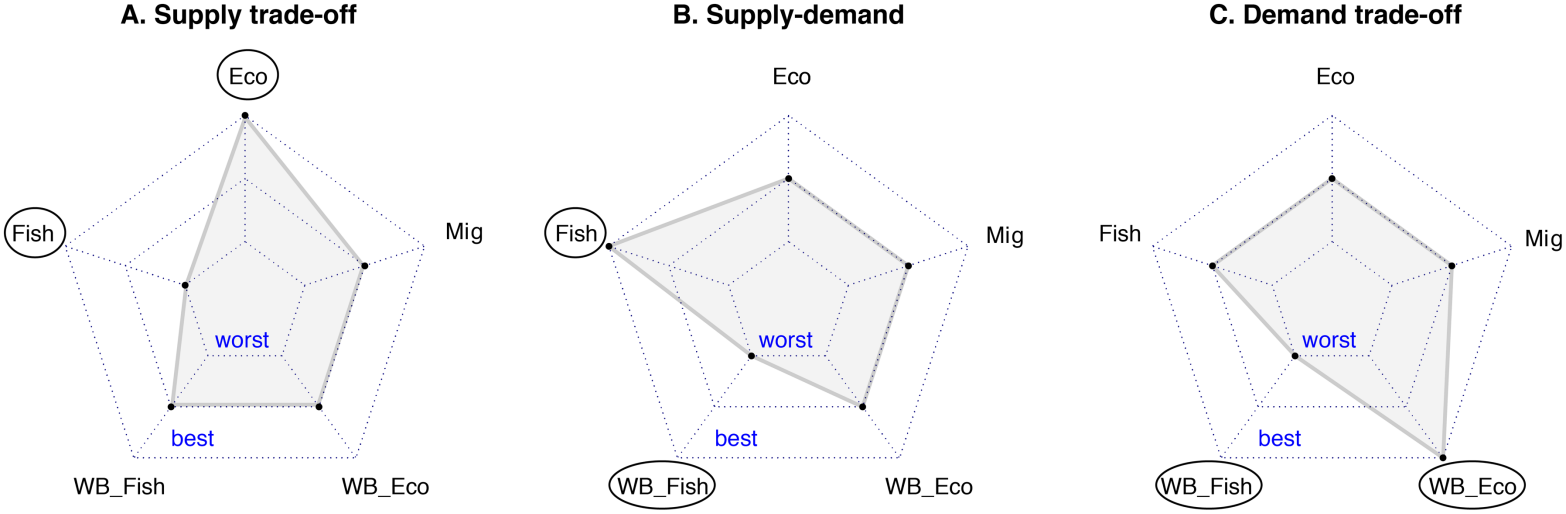
Visual representations of how the three conceptual trade-offs (as identified by Mouchet *et al*. (23)) may appear across the seven outcomes assessed in our study. Each example radar plot (A,B,C) shows all five focal outcomes (ecosystem health, migratory species, fishery resources, well-being of user groups (e.g., fishers), and well-being of users of the ecosystem (e.g., coastal residents, tourists), with the inner-most band representing a decline and the outside line representing an increase (indicated with ‘worst’ to ‘best’ on the radar plot). Key outcome trade-offs have been circled to aid understanding of the trade-off typology and how it applies to our data. Outcome abbreviations used in radar plot: Eco = ecosystem health change; WB_Eco = well-being change of the user of the ecosystem health indicator; WB_Fish = well-being change of the user of the fisheries indicator; Mig = migratory species change; Fish = fisheries change. **A**: Supply trade-off: ecosystem health improving, but fisheries declining (or vice versa; conservation versus use). **B**: Supply-demand trade-off: fisheries improving, but well-being of a user (fisher) declining (or vice versa). **C**: Demand trade-off: differentiated impacts in the well-being of different users, with a well-being decline of a user dependent on fisheries, and a well-being improvement of a user dependent on ecosystem health (e.g. tourism) (or vice versa).

Potential trade-offs were identified using radar plots in R (version 3.2.2 [30]) and the fmsb package [31]. Radar plots provide visualisations of multivariate data in a simple two-dimensional chart. First, radar plots were analysed visually to identify potential trade-offs where one outcome was stable or increasing and another was declining, as depicted in Fig 1. Potential supply trade-offs are indicated by different outcomes in ecosystem health, fisheries, and/or migratory species. Potential supply-demand trade-offs are indicated by differences between outcomes for ecosystem health or fisheries, and the well-being of associated user groups. Potential demand trade-offs are indicated by differences in the well-being of different user groups. Although variation in outcomes is indicative of a potential trade-off, these may be coincidental rather than causal. As a result we complement our analysis of outcomes with a qualitative analysis of the plausibility of a causal mechanism linking the two outcomes (Table 3) to understand if different outcomes were potentially causal (trade-off) or merely coincidental (divergent outcomes).

**Table 3:** Mechanisms that may give rise to trade-offs, including description and examples from the literature.

Trade-off Mechanisms	Description	Example
Management priorities	Management decisions prioritize certain objectives, and invest more in associated activities. Applies to supply, supply-demand, and demand trade-offs.	MPAs commonly prioritize management that benefits ecosystems, resulting in the ‘classic’ conservation trade-off between protection or use of resources (e.g., [32]). Lack of management actions for some species can result in lack of recovery (e.g., migratory species [33]).
Everyday resource use decisions	Trade-offs arise between extraction and short-term well-being or resource conditions and long-term sustainability. Applies to supply-demand trade-offs.	Overfishing is associated with increases in (short-term) well-being at the expense of resource conditions [34]. Conversely conservation of harvested resources can lead to improved resource conditions at the expense of the short-term well-being of actors that depend upon them [35].
Externality of resource use	Some trade-offs occur as an unintended consequence of resource use where the exploitation of one resource has impacts on others. Applies to supply trade-offs.	Some fishing gears cause destruction or alteration of habitats that other natural resources depend on. Similarly, fishing can have incidental mortality of non-target species that are targeted in a different fishery (e.g., [36]).
Biophysical relationships	Conditions of one environmental good or service are dependent on the conditions of other environmental goods or services. Applies to supply trade-offs.	Trophic cascades can occur as a response to protection [37]. Health and abundance of seabirds depends upon abundance of forage species [38].

We categorised four types of causal mechanisms that can lead to trade-offs (Table 3): 1) deliberate *a priori* management decisions to prioritize some outcomes over others (32), or the allocation of finite resources to some activities over others (33); 2) everyday resource use decisions by resource users that influence well-being and resource conditions [39, 40]; 3) unintended consequences of resource use where the exploitation of one resource has a direct impact on others (e.g., by-catch) [41]; 4) indirect consequences that occur when two or more resources are connected via biophysical relationships or ecosystem processes (e.g., food webs) [42]. This last type of trade-off mechanism is less visible than others and can take longer to manifest.

## Results

**Fig 2:**
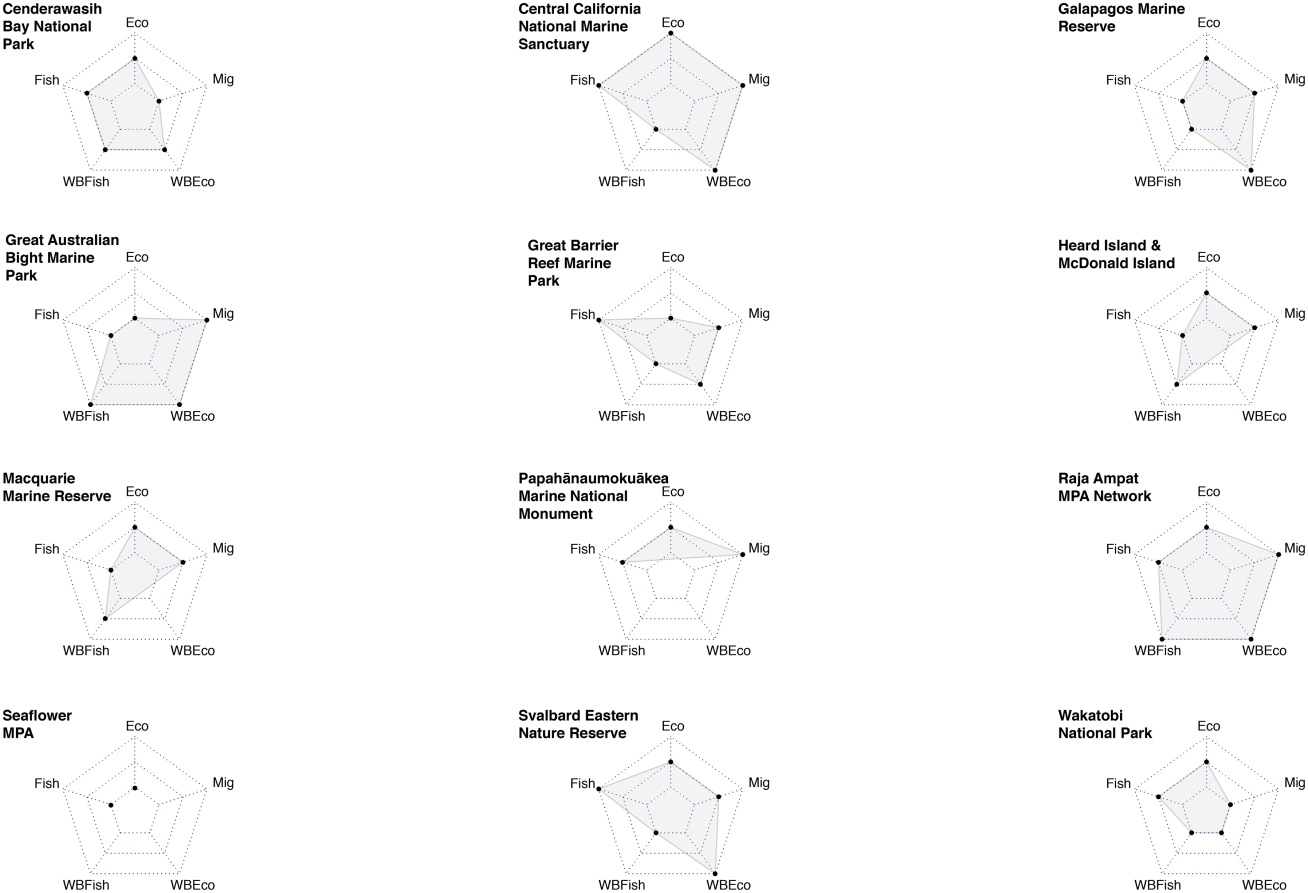
Radar plots of all outcomes for each case study. Inner line is declining status, middle line is same or mixed effects, and outer line is increasing status. Missing data (either where there was no user so an outcome was not appropriate, or no data present) were not plotted as points on the radar chart and the lines connect the points where data were present. Outcome abbreviations used in radar plot: Eco = ecosystem health change; WBEco = well-being change of the user of the ecosystem health indicator; WBFish = well-being change of the user of the fisheries indicator; Migratory = migratory species change; Fish = fisheries change.

We found varying outcomes in all LMPAs (Fig 2). Radar plots revealed potential trade-offs in nine of the twelve cases. These included seven cases with possible supply trade-offs, eight with possible supply-demand trade-offs, and four with possible demand trade-offs. Evaluation of trade-off mechanisms in these cases found evidence to explain three supply trade-offs, four supply-demand trade-offs, and one demand trade-off. Trade-offs appeared to develop as a result of *a priori* management decisions, and everyday resource use decisions. Divergent outcomes were attributed to a variety of external factors such as international conservation measures, and land-based pollution.

### Supply trade-offs among ecological outcomes

In the seven cases with differences in ecological outcomes, three involved changes in migratory species and other environmental outcomes (fisheries and ecosystem health). In Wakatobi National Park and Cenderawasih Bay National Park, the migratory species (green turtle) was declining, whereas other ecological indicators remained stable. This trade-off is likely the result of management decisions to prioritize other aspects of the environment in a context of limited resources. The governance system does not appear to be adequate for vulnerable species, particularly green turtles where historical overexploitation in Indonesia has increased their vulnerability to incidental take in small-scale fisheries [43]. Conversely, in the Great Australian Bight Marine Park, the migratory species (southern right whale) was increasing, while other ecological indictors were declining. This MPA protects calving grounds for the southern right whale, but the species is also benefiting from wider protection methods—primarily the global moratorium on whaling led by the International Whaling Commission, indicating a divergent outcome rather than trade-off.

Three cases had differences between the fishery and the other ecological outcomes (ecosystem health and migratory species), but only one of these appeared to involve a trade-off mechanism. In the Galapagos Marine Reserve, regulations in the brown sea cucumber were not enforced and the species is now considered commercially extinct [44]. This fishery faced strong demand from globalised markets, providing strong incentives for fishers to exploit resources for short-term economic gain. The fishery had also previously experienced high levels of conflict (see [45]), which contributed to management decisions to avoid potential conflicts with fishers, rather than ensuring the sustainability of invertebrate fisheries. In contrast there is no reason to suspect that declines in the Sub-Antarctic fisheries (Heard and McDonald, Macquarie) are related to the other ecological outcomes because the decline reflects management decisions to begin harvesting formerly unexploited stocks rather than a trade-off between priorities. Nonetheless it is worth noting that these fisheries have adopted strict management measures, including technological and operational requirements, 100% observer coverage, and seabird bycatch limits to minimise trade-offs between fisheries and migratory seabirds. In the remaining case—the Great Barrier Reef Marine Park—the different outcomes were between ecosystem health change and the fisheries and migratory species changes. In this MPA, coral cover has declined significantly, despite the re-zoning of the marine park in 2004, mainly due to land-based impacts and climate change, which are not directly within the scope of the MPA governance system [46].

### Supply-demand trade-offs between ecological and social outcomes

In the eight instances where social and ecological outcomes differed, seven involved fisheries and dependent fishers. Four of these appear to be trade-offs, driven by management decisions and everyday resource use decisions. For example, in the Great Barrier Reef Marine Park, extensive no-take zones (33% of the MPA) have contributed to increased fish biomass [47], but have increased costs for fishers who remained in the fishing industry by reducing the availability of fishing grounds [48]. In Wakatobi National Park, the Bajau are the main users of marine resources and fishing is central to their culture, but they have been marginalised by state and NGO initiatives in the MPA [49]. Likewise, in the Central California National Marine Sanctuaries, new gear restrictions, permits and mandatory on-board observers, have affected the economic viability of groundfish fisheries.

### Demand trade-offs among social outcomes

Potential demand trade-offs were observed in four cases. Three involved different user-groups (Svalbard Eastern Nature Reserves, Central California National Marine Sanctuaries, Galapagos Marine Reserve), and one involved the same user-group but different ecological resources (Great Barrier Reef Marine Park). However, only one of these appears to be a trade-off (Galapagos Marine Reserve). Tourism has been actively promoted and is increasing in both the Galapagos Marine Reserve and Svalbard Eastern Nature Reserves, while fishers have faced declines in well-being. In the Galapagos Marine Reserve economic incentives were provided to sea cucumber fishers after the collapse (and closure) of the fishery to encourage alternative livelihoods related to tourism [50]. In contrast, there is no direct link between the shrimp fishery and tourism in the Svalbard Eastern Nature Reserves, as declines in fisher well-being reflect decreases in the value of shrimp landings. Other examples of divergent outcomes include the Central California National Marine Sanctuaries, where academic research has benefited from increased long-term commitments to research within the MPA, infrastructure and funding opportunities. Conversely, the restrictions placed on groundfish fishermen have led many to exit the fishery, but because management plans of the Sanctuaries do not aim to reduce the number of California groundfish fishermen and they are not in direct competition for resources with academic researchers, it is considered a divergent outcome.

### Lack of evidence of trade-offs

Three cases lacked evidence of all types of trade-offs. In both Papahānaumokuākea Marine National Monument and Raja Ampat MPA network, all ecological outcomes were stable or improving, while evaluated social outcomes in the Raja Ampat MPA network were also improving [51]. No user group was coded for the Papahānaumokuākea Marine National Monument because the whole area is completely no-take and there is no significant direct resource user. However, indigenous Hawaiians are now able to access the area for cultural purposes but currently no data exist for this use. Conversely the Seaflower MPA had declines for two ecological outcomes, but lacked data for the other outcomes, meaning a comprehensive assessment of trade-offs in this case was not possible.

## Discussion

The establishment of LMPAs continues at a rapid pace as governments around the world seek to meet ambitious international targets, manage risks associated with climate change [52], and facilitate the management of trade-offs across wide ranging social and environmental objectives (7, 8). However, studies about the ecological and social outcomes of LMPAs have been limited to date, with more emphasis on hypothesized rather than realized outcomes [7–12]. Here we provided clear evidence that size alone is insufficient for avoiding trade-offs or important divergent outcomes in MPAs. Indeed, we observed considerable variability in the social and ecological performance of individual LMPAs, and in several cases, were able to link divergent outcomes through a plausible causal mechanism, which we considered a trade-off. Furthermore, we observed supply trade-offs among resources, demand trade-offs among user groups, and supply-demand trade-offs between user groups and resources despite our relatively small sample of 12 LMPAs. Collectively, these findings should encourage managers and researchers to pay close attention to trade-offs, even within LMPAs.

### Trade-offs in LMPA management

We found the typology of trade-offs [23] to be useful in conceptualizing and assessing trade-offs between different ecological resources (supply trade-offs), trade-offs between ecological resource conditions and the well-being of resource users (supply-demand trade-offs), and trade-offs between the well-being outcomes of different resource users (demand trade-offs). Scrutinizing these potential trade-off relationships enables a nuanced understanding of the complexities of who and what benefits from LMPAs. We suggest that LMPAs planners and managers should consider trade-offs that are likely to occur and look to monitor across a range of social and ecological outcomes so that any negative impacts can be foreseen and managed.

We found evidence of all three types of trade-offs in our sample of 12 LMPAs, highlighting the importance of considering and mitigating trade-offs where possible. Much like smaller MPAs, supply-demand trade-offs were observed frequently as managers face challenges in balancing fisheries catches (and hence impacts on fishers) with the long-term sustainability of fish stocks. These types of trade-offs tend to develop when policymakers prioritize conservation at the expense of the well-being of user groups [53, 54], or alternatively, allow resources to decline in order to maintain or enhance the social and economic well-being of user groups [34, 55]. However, it has also been noted that such trade-offs may be short in duration [56] as fish stocks either recover and contribute to ‘win-win’ outcomes, or continue to decline with subsequent impacts on the well-being of user groups (i.e. ‘lose-lose’ outcomes). In fact, LMPAs may be more susceptible to supply-demand trade-offs if fishers face difficulties in accessing the spill-over benefits that commonly develop in smaller MPAs [57–59]. Supply-demand trade-offs involving tourism and their consequent disturbance of marine vertebrates have also been observed in smaller MPAs [60, 61]. Although tourists appear to have had a limited impact on outcomes in our sample of LMPAs, tourism can be a contributor to conservation [62], but there are growing concerns about the potential impacts of increases in marine tourism, particularly in remote areas [63].

Supply trade-offs between resources and demand trade-offs between user groups have typically received less attention in the conservation literature than supply-demand trade-offs. Nonetheless, our study suggests that such trade-offs are salient in the context of LMPAs. First, supply trade-offs among resources appeared to develop when managers faced decisions about how to allocate limited resources (time, effort, political capital) among different ecological resources. For instance, managers in the Galapagos Marine Reserve appear to have allowed the collapse of brown sea cucumber in order to maintain support for broader conservation initiatives. Supply trade-offs in smaller MPAs, meanwhile, often involve endangered species, which are neglected by managers because of the size and scope of the conservation challenge [33]. Indeed, many endangered species are highly sensitive to incidental take (bycatch) as a non-target species, in both industrial [64] and small-scale fisheries [65], making it difficult to avoid impacts without affecting the well-being of fishers. Second, demand trade-offs between user groups also occurred in the Galapagos Marine Reserve, where tourism interests have generally benefited at the expense of fishers, problems which have also been found in smaller MPAs [66–68]. Finally, although we found evidence of all three types of trade-offs within LMPAs, we also found divergent outcomes, which appear to be driven by larger-scale social, economic, and ecological drivers, such as international market pressures, international treaties, land-based activities, and climate change. Thus, in accessing trade-offs in a management context, it is critical to be cognisant of the potential of divergent outcomes.

Increasingly climate change impacts will also influence how trade-offs manifest [69]. While climate change will likely negatively affect many ecological resources and the well-being of their users, some impacts and associated adaptation/management responses will enable some ecological resources or user groups to benefit (e.g., squid fisheries in the North Sea [70]). Indeed, climate change is increasingly used to justify management decisions that prioritise particular supply-demand and demand trade-offs, for instance designation of no-go areas to enhance ecological recovery post-impact, or prohibition or modification of particular fishing gears thought to impact important ecological functional groups (e.g., [71]).

### Study limitations

Our research was constrained by several limitations. First, variability in levels of monitoring and reporting on the 12 cases influenced the coding of cases and analysis of trade-offs. In some cases, such as the Seaflower MPA, we were unable to code certain outcomes, precluding the analysis of some trade-offs. Additionally, the availability of information in other cases (e.g., only on certain natural resource outcomes) may have affected our results by influencing the structure of our cases. Second, our focus on changes to resources and user groups over the time period examined—rather than looking at status as it currently stands—was constrained by the relative paucity of data showing changes to resources and user group well-being over time. However, we felt that changes over time would more accurately reflect the effectiveness of the LMPAs, whereas status alone would not be as informative without reference points.

Our decisions about which user groups and resources to code were based upon a preliminary analysis of the availability of information to code outcomes, while also facilitating comparison across cases. As a result, the Great Barrier Reef case grouped all commercial fishers into a single user group and found that their overall well-being had declined between 2000 and 2012. However, a finer grained study that differentiated groups on the basis of target species or fishing gear may have resulted in more specific understanding of types of outcomes or trade-offs. Also, a declining status in a resource is not necessarily a reflection of ineffective management. For instance, the Patagonian Toothfish fishery at Macquarie Island has declined steadily since the fishery was established in 1994, but is generally considered sustainable by experts, the Australian Government and Marine Stewardship Council [72, 73]. Therefore, although our analysis allows us to confirm the existence of trade-offs in terms of relative changes in social and ecological outcomes in LMPAs, we are unable to provide a strong empirical assessment of the frequency and distribution of different types of trade-offs that may be occurring within LMPAs. Future studies should seek to address this gap by investigating a greater diversity of species and user groups to better understand the prevalence of trade-offs in LMPAs.

## Conclusions

Notwithstanding the limitations discussed above, our study provided clear evidence of several different types of trade-offs between users and resources in LMPAs, and highlighted the influence of external factors in contributing to divergent outcomes. We provided examples of potential trade-offs that can be used to guide discussions and plans for current and future LMPAs. More research in this area can provide opportunities to improve the management of LMPAs, including how considerations of location (remoteness), zoning (e.g. no-take versus multiple use zones), the overall state of the ecosystem (pristine versus degraded) influence trade-offs, and how climate change might affect trade-offs. Recognising that trade-offs can occur, even in these large areas, facilitates managing them in ways that can include specific actions for affected user groups or focal species.

## Supporting information

S1 Fig Map of the 12 LMPAs that met our criteria to be included in the study. Information includes MPA name, country of origin, date of designation, and total size.
(TIF)
